# Development and juvenile anatomy of the nemertodermatid *Meara stichopi* (Bock) Westblad 1949 (Acoelomorpha)

**DOI:** 10.1186/1742-9994-11-50

**Published:** 2014-07-07

**Authors:** Aina Børve, Andreas Hejnol

**Affiliations:** 1 Sars International Centre for Marine Molecular Biology, University of Bergen, Thormøhlensgate 55, 5008 Bergen, Norway

**Keywords:** Nemertodermatida, Acoelomorpha, Development, Muscle development, Neurogenesis, Cleavage, Cell lineage

## Abstract

**Introduction:**

Nemertodermatida is the sister group of the Acoela, which together form the Acoelomorpha, a taxon that comprises bilaterally symmetric, small aquatic worms. While there are several descriptions of the embryology of acoel species, descriptions of nemertodermatid development are scarce. To be able to reconstruct the ground pattern of the Acoelomorpha it is crucial to gain more information about the development of several nemertodermatid species. Here we describe the development of the nemertodermatid *Meara stichopi* using light and fluorescent microscopic methods.

**Results:**

We have collected *Meara stichopi* during several seasons and reconstruct the complex annual reproductive cycle dependent on the sea cucumber *Parastichopus tremulus*. Using common fluorescent markers for musculature (BODIPY FL-phallacidin) and neurons (antibodies against FMRFamide, serotonin, tyrosinated-tubulin) and live imaging techniques, we followed embryogenesis which takes approximately 9–10 weeks. The cleavage pattern is stereotypic up to the 16-cell stage. Ring- and longitudinal musculature start to develop during week 6, followed by the formation of the basiepidermal nervous system. The juvenile is hatching without mouth opening and has a basiepidermal nerve net with two dorsal neurite bundles and an anterior condensation.

**Conclusions:**

The development of *Meara stichopi* differs from the development of Acoela in that it is less stereotypic and does not follow the typical acoel duet cleavage program. During late development *Meara stichopi* does not show a temporal anterior to posterior gradient during muscle and nervous system formation.

## Introduction

The clade Nemertodermatida comprises only nine described species of small, completely ciliated, exclusively marine, hermaphroditic worms that live mostly in interstitial habitats [1,2]. Nemertodermatids possess a medio-ventral mouth that is the sole opening to the epithelial, sack-like gut. The nervous system is located basiepidermally, and all nemertodermatid species possess a characteristic double-statocyst or gravitational sensory organ [3]. Nemertodermatida and Acoela (together forming the Acoelomorpha [4]) have recently gained attention because of their disputed phylogenetic position, which greatly impacts our understanding of the evolution of animal body plans [5,6]. These rather simple worms have been placed as sister group to all remaining Bilateria [7-14] – in some studies as separate branches [15,16] - and thus helpful to understand the evolutionary transition of the cnidarian-bilaterian stem species into the bilaterian stem species [6]. Alternative hypotheses place acoelomorphs either as sister group to all remaining deuterostomes [10] or as sister group to the Ambulacraria (Echinodermata + Hemichordata) [10]. In both latter cases, the lack of some morphological features in acoelomorphs, such as nephridia and gill slits, would be interpreted as independent losses [17]. Nemertodermatids play a key role for determining the direction of character evolution in the Acoelomorpha [18]. Nemertodermatids share plesiomorphic characters such as a basiepidermal nervous system, monoflagellate sperm, and an epithelial gut [4,18,19] and lack acoel novelties, including a subepidermal brain and parenchymal tissues [18,19]. Nemertodermatids share these characters with members of the Xenoturbellida, a possible sister group of the Acoelomorpha [9,10,13]. A thorough comparison of the morphology and development of xenoturbellids, nemertodermatids and acoels is essential to gain a deeper insight into the ancestral character states of this taxon and the changes during cell type and organ system evolution.

*Meara stichopi*[20] and *Nemertoderma westbladi*[21] are the two most accessible nemertodermatid species, and both species can be collected relatively easily from the field. Embryos from both species can be obtained for developmental studies (present study and [22]), but detailed descriptions of the embryology are still missing. Here we describe the development of *Meara stichopi* and compare it with previous studies of acoel and nemertodermatid embryos.

## Results

### The annual reproductive cycle of *Meara stichopi* and presence in the host *Parastichopus tremulus* (Gunnerus, 1767)

**Figure 1:**
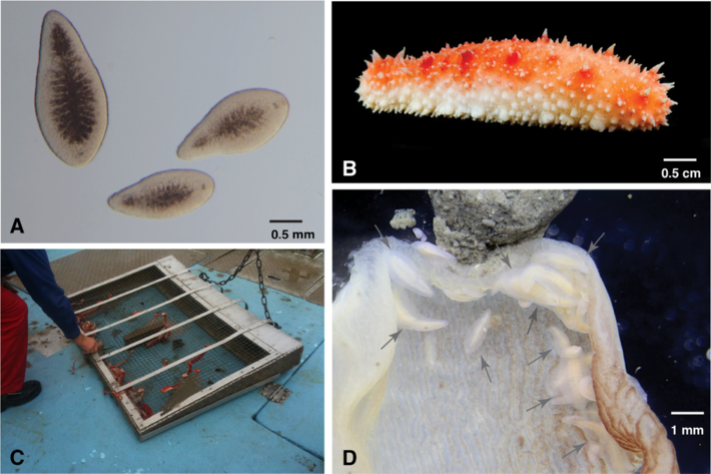
**Collection of *Meara stichopi*. A)** Three individuals of *Meara stichopi* from a collection in June. Individuals are not gravid and the size range is between 1–2 mm. **B)** Sea cucumber *Parastichopus tremulus*, the host of *M. stichopi* (photo courtesy of Mattias Ormestad, kahikai.org, anterior to the right). **C)** The “Schander sled”, after dredging in 250 m depth in the Lysefjorden. Red *P. tremulus* sea cucumbers visible in the mesh. **D)** Opened foregut of *P. tremulus* with adult *M. stichopi* (arrows). Gut content visible on top.

Our sampling over four years revealed novel insights into the life cycle of *Meara stichopi* and its seasonal reproduction. As reported in the species description [20], *M. stichopi* is mainly found in the first 3 cm of the foregut of its host, the sea cucumber *Parastichopus tremulus* (Figure 1). We observed that *P. tremulus* collected on coarse sandy bottoms (e.g. Sognefjord, Hardangerfjord) did not contain any *M. stichopi*, possibly because the sand grains prevent *M. stichopi* from attaching to the foregut wall. We observed *M. stichopi* only inside sea cucumbers living on muddy bottoms, often in large numbers (up to 100–200 individuals) (Figure 1D), where they are mainly affiliated with the gut wall and largely absent from the gut content. We have observed that most individuals are oriented with the mouth directed toward the gut content.

**Figure 2:**
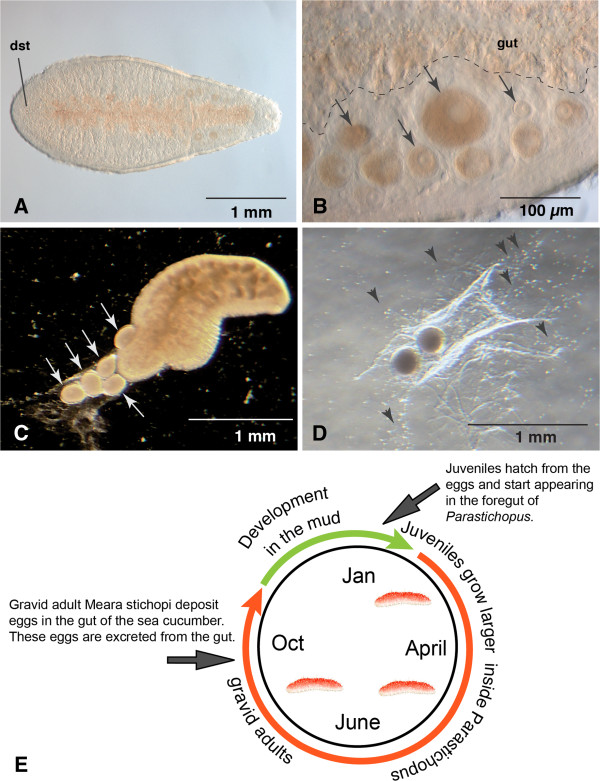
**Egg deposition of gravid *M. stichopi *and model of annual life cycle. A)** Gravid adult of *Meara stichopi* collected in September. The characteristic double statocyst (dst) at the anterior end is indicated. **B)** Close-up of oocytes in different stages of the adult (black arrows). Note that smaller oocytes are located more distally than the large oocytes. Anterior to the left, the gut is labeled with the dotted line. **C)** Five eggs (white arrows) deposited in a glass bowl by an adult *M. stichopi* oriented with the anterior end to the embryos. **D)** Two eggs in jelly deposited on the bottom of the glass bowl. Small dots surrounding the eggs are motile spermatozoa (arrowheads). **E)** Model for annual cycle of *M. stichopi*. According to the model, fertilized eggs exit the sea cucumber through the gut and develop between 9–12 weeks in the sediment. After hatching, the juveniles initially do not have a mouth opening and survive from the nutrients of the yolk. We presume the juveniles are ingested by the sea cucumbers, where they are able to adhere to the foregut of the sea cucumber, and live as commensals. The juveniles grow to adults over the next months and start to become gravid in August-October. Fertilization occurs in the foregut of the sea cucumber and eggs are deposited, probably exiting the gut of the sea cucumber through the anus. The adults disintegrate after egg deposition and are digested by the sea cucumber. The approximate variation of development covers a period of three months, which also includes the time window when gravid adults are observed to deposit eggs.

We have detected an annual pattern of presence and size variation of *M. stichopi* in the gut of the host. With few exceptions *M. stichopi* was completely absent from the gut of the sea cucumbers between the months of November and February (Figure 2E). In samples from mid March onward, small individuals (150 μm long) are present in the foregut of the sea cucumber, initially in small numbers. The number of individuals in the foregut increased to 150–200 over the course of the following months. From April to October, individuals observed in the foregut are larger in size, measuring up to 5 mm in length (Figure 2A). From August on, we observed different staged oocytes in the gonads of the adults, with the matured oocytes located close to the gut tissue (Figure 2B). Nemertodermatids do not possess gonads that are surrounded by epithelia. The number of individuals slowly decreased from August until November, when *M. stichopi* is no longer observed in the sea cucumber. When searching for *M. stichopi* during the end of October and examining the entire gut of the sea cucumber, we found partially digested large individuals in the midgut. We have never found living *M. stichopi* in this gut region nor did we find any embryos anywhere in the digestive tract of the sea cucumber. From these findings, we surmise that *M. stichopi* has an annual life cycle with embryonic and juvenile stages outside of the sea cucumber (see Figure 2E and Discussion).

### Reproduction and fertilization

**Figure 3:**
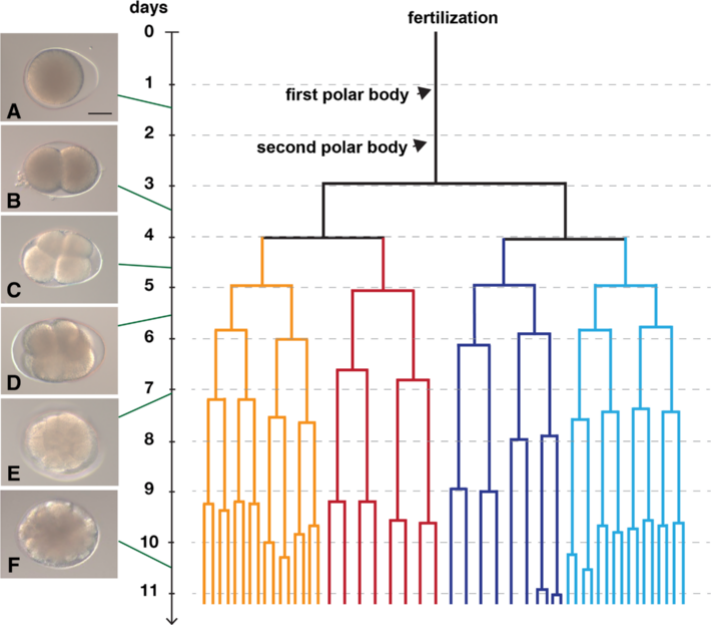
**Timing of the cell divisions of an embryo of *M. stichopi *up to the 50**-**cell stage.** Cell divisions of a single embryo recorded with time-lapse microscopy. The lineage of the vegetal blastomeres indicated with dark blue and dark red branches, the animal blastomeres in light blue and orange branches. The duration of the cell cycle increases during the course of development from 24 hours to 3 days. **A**-**E)** Fertilized egg and cleavage stages imaged with Nomarski optics. **A)** Fertilized egg with egg shell, **B)** 2-cell stage. **C)** 4-cell stage, **D)** 8-cell stage **E)** 16-cell stage, **A**-**E**, same embryo. **F)** different embryo in a 48-cell stage. Scale bar: 30 μm.

Gravid animals begin to deposit eggs following their transfer to small glass bowls (Figure 2C, D). Since the only body openings are the mouth and male gonopore, immediately following egg deposition, we fixed individuals (n = 10) and labeled them with BODIPY FL-phallacidin to examine possible ruptures of the musculature. However, we could not detect any ruptures in the muscle net and presume that eggs are deposited through the mouth, although we have never observed egg deposition directly. Gravid individuals deposit up to six oval, yolky eggs of ~100 μm length into a mucus-sheath on the bottom of the glass bowl (Figure 2C, D). We often observed motile spermatozoa around the oocytes (Figure 2D). Immediately after being deposited, oocytes are irregularly shaped and lack an eggshell. These eggs become spherical and develop a clear, oval-shaped eggshell, likely a result of fertilization (Figure 3). Against previous assumptions [20], we speculate that fertilization is external due to the observance of sperm near the oocytes, but we have not determined if the sperm originated from the same or different individuals.

### Cleavage and gastrulation

**Figure 4:**
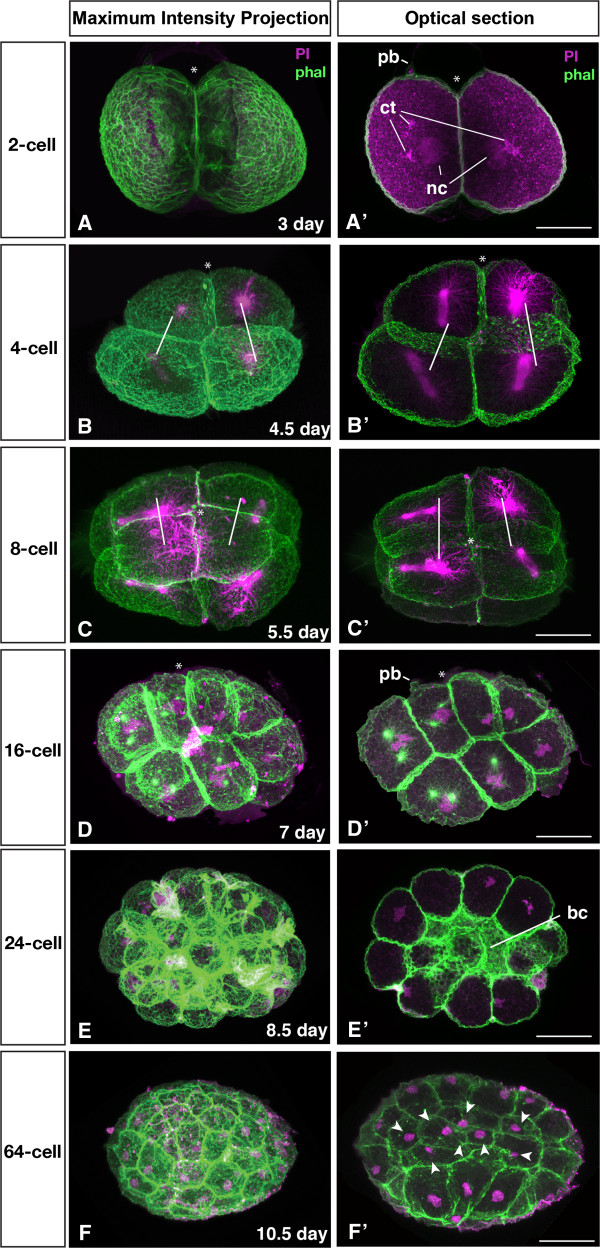
**Early cleavage pattern of *Meara stichopi *embryos.** Nuclear labeling with Propidium Iodide (magenta), cell cortices and spindle with BODIPY FL-Phallacidin (green) Left row Maximum Intensity Projections, right row optical sections. **A)** 2-cell stage (3 days after fertilization). One of the polar bodies (pb) is visible at the animal pole. **A’)** shows an optical section through the same embryo. Propidium iodide is labeling the chromatin in the nucleus (nc) as well as the centrosomes (ct). Both blastomeres are equal in size. **B)** After 4.5 days, the 4-cell stage has large blastomeres at the vegetal pole, and two smaller daughter blastomeres at the animal pole. **B’)** shows a section of the embryo in B). The spindles are arranged for the future direction of cell division. **C)** After 5.5 days the 8-cell stage is composed out of four larger cells at the vegetal pole with four blastomeres at the animal pole. **C’** shows an optical section of the embryo of C), with spindles arranged to the future plane of division. **D)** 16-cell stage reached 7 days after fertilization. The size differences between the blastomeres are less prominent and the arrangement is variable. **D’)** BODIPY FL-phallacidin labeled cell borders as well as the centrosomes, while the chromatin is labeled by propidium iodide. **E)** 24-cell stage after 8.5 days. **E’)** shows a median section of the embryo shown in E). The blastocoel is bordered with the phallacidin labeled cell cortex of the outer blastomeres. **F)** 64-cell stage 10.5 days after fertilization. **F’** shows the cells that have been internalized (blastomeres labeled with arrowhead) during the transition from the 24 to the 64 cell stage. Sister blastomeres are connected by white bars, animal pole is indicated with an asterisk. Scale bar: 30 μm.

The development of *M. stichopi* can be characterized as fairly slow. When cultured at 6-8°C, embryos developed for 9–10 weeks until the hatching of the juvenile. Our observations using light microscopy and 4D-microscopy show that the zygotes extrude two polar bodies after fertilization, with the first cleavage observed three days after egg deposition (Figure 3). The first polar body is observed approximately 24 hours after fertilization (Figure 3). The polar bodies mark the animal pole of the embryo, however they are not visible later in development, making it difficult to orientate embryos in later stages. The first cell division takes place about 24 hours after the 2^nd^ polar body has been given off and is equal and meridional (Figures 3 and 4A). BODIPY FL-phallacidin labels the F-actin of the cell cortex of the blastomeres and propidium iodide stains nucleic acids of the nucleus and cytoplasm, as well as the centrosome (Figure 4). The second cleavage is equatorial and unequal, resulting in two smaller animal micromeres and two vegetal macromeres. The micromeres are not centered on top on the macromeres, but are instead slightly shifted in relation to the animal-vegetal axis of the embryo (Figures 3 and 4B). The interval between first, second and third cleavage is about 24 hours (Figure 3). At the 8-cell stage, the planes of cell division are all equatorial and equal, forming a tier of four blastomeres at the animal pole and four larger blastomeres at the vegetal pole (Figure 4C). The four animal blastomeres are situated directly on top of the vegetal blastomeres and not between the vegetal blastomeres as it is the case in spiralian embryos. The following cell divisions are equal and asynchronous (up to 24 hours apart, see Figure 3) and the cleavage planes vary in their angle between the blastomeres. In general, the cleavage planes are parallel to the surface of the embryo producing equally sized blastomeres (Figure 4D, E). The durations of the cell cycles vary from less than 24 hours up to 43 hours (Figure 3). The live recording of embryos reveals that the cell cycles of the vegetal blastomeres are longer compared to the cell cycles of the animal blastomeres (Figure 3). At the 24-cell stage, individual cells can only be identified via cell tracing (Figure 4E) but not based on their size or shape. A small blastocoel is visible by the phallacidin labeling of the cell cortices (Figure 4E’). Nine days after fertilization, two or more cells are located internally, indicating the beginning of gastrulation (n = 6). After a round of cell divisions, several more cells are now located inside the embryo (Figure 4F). The internalized cells appear smaller than the outer cells (Figure 4F’). The internalized blastomeres are probably the endomesodermal precursor cells.

### Further development and morphogenesis

**Figure 5:**
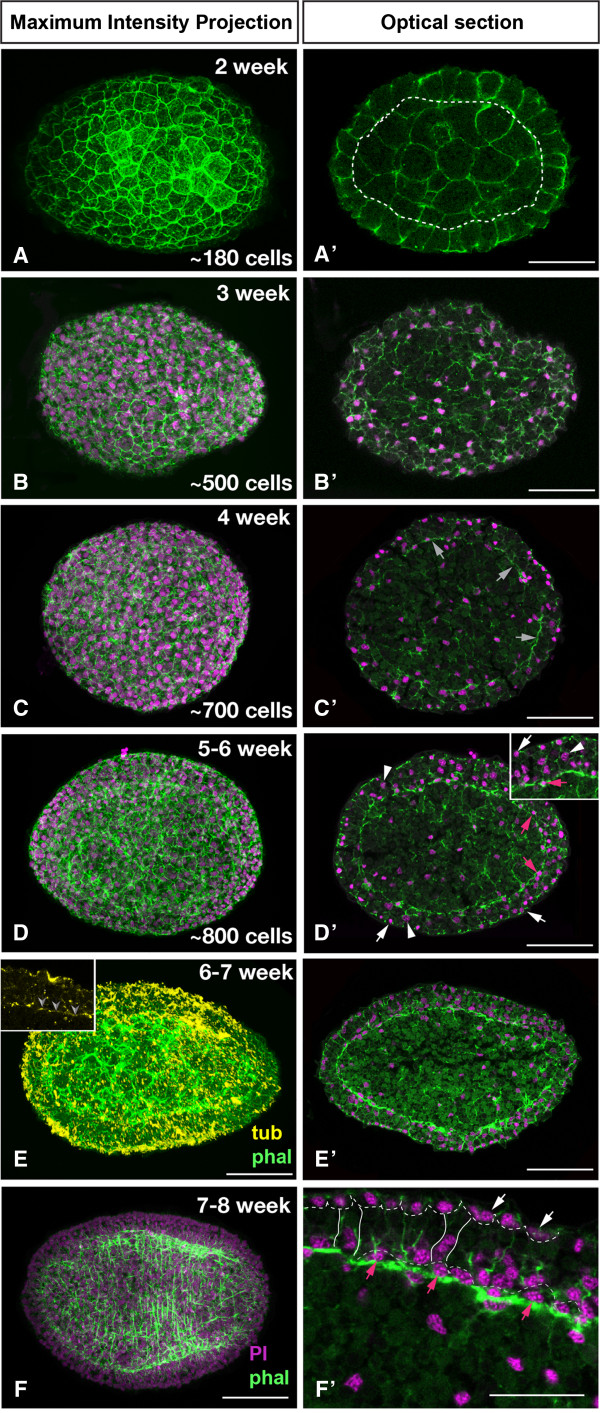
**Later development of *M. stichopi *embryos including muscle formation.** Nuclear labeling with Propidium Iodide (magenta), muscle fibers with BODIPY FL-Phallacidin (green) and anti-tyrosinated tubulin (yellow). Left row Maximum Intensity Projections, right row optical sections. **A)** Embryo two weeks after fertilization with ~180 cells labeled with BODIPY FL-phallacidin. **A’)** shows the inner cell mass (encircled by dotted line) in an optical section of the embryo shown in **A)**. **B)** Embryo with ~500 cells three weeks after fertilization. **B’)** shows the nuclei close to the cell membrane of each cell. **C)** 4-week old embryo composed out of approximately 700 cells. **C’)** Optical section of **C)**, with actin filaments visible that indicate the beginning of the formation of muscle fibers (arrows). **D)** Dorsal view on 5–6 week old embryo composed out of ~800 cells. The actin fibers of the myocytes are visible in all areas of the embryo. **D’)** Optical section of **D)** with subepidermal signal of BODIPY FL-phallacidin visible in multiple areas of the embryo. **E)** The labeling of tyrosinated-tubulin in 6–7 week old embryo shows the cilia in the epidermis of the embryo (yellow), dorsal view. The phallacidin labeling of the musculature has become more prominent but is still irregular. **E’)** Optical cross section through another embryo in the same age as **E)** The propidium iodide labeled nuclei and the musculature, dorsal view. **F)** The 7–8 week old embryo shows regularly arranged muscle fibers corresponding to the future pattern of the ring-musculature. **F’)** most nuclei are located at the apical pole of the epidermal cells (white arrows). Other nuclei are located also at the base of the epidermis (red arrows), likely the nuclei of the neural precursors of the basiepidermal nerve net. Scale bar 30 μm in all images except F’ 10 μm.

Approximately two weeks after fertilization, the embryo is composed of approximately 180 cells, with an inner cell mass of larger blastomeres that are surrounded by an outer layer of smaller non-epithelial cells (Figure 5A). The internal cells are larger in size than the outer cells. This may indicate that the inner cells undergo fewer divisions than the outer cells (Figure 5A’). After three weeks, the embryo is composed out of approximately 500 cells (Figure 5B). Interestingly, the nuclei are located at the margin of each cell in an irregular pattern, suggesting planar cell polarity is not yet established (Figure 5B’). Four weeks after fertilization, the embryo is composed out of approximately 700 cells (Figure 5C). The optical section through the center of the embryo shows that some of the nuclei of the outer cell layer are located at the apical side of the cells (Figure 5C’). Muscle fibers become visible just below the outer cell layer and reveal the formation of actin bundles of the musculature, indicating the epithelial character of the outer cell layer (Figure 5C’). Five to six weeks after fertilization, more muscle fibers become visible and are arranged in an irregular network that extends along the anterior-posterior axis (Figure 5D, D’). The nuclei of the outer layer of the embryo are in three different positions: I. In an apical position, indicating the development of the flat, multiciliary epidermal cells (Figure 5D’, white arrows); II. In the center of cylindrical cells that form the main epidermal cell layer (Figure 5D’, arrowheads); III. At the base of the epidermal layer forming the differentiating neurons of the future nerve net (Figure 5D’, red arrows). Additional nuclei are located below the base of the outer cell layer and are affiliated with the muscle fibers. Phallacidin labeled fibers are also visible in the internal region of the embryo, indicating that cross-musculature begins to form (Figure 5D’). Six to seven weeks after fertilization, the network of muscle fibers is more dense, but still irregular (Figure 5E). The anti-tubulin staining indicates that the epidermal cells begin to form cilia (Figure 5E). Nerve fibers are also visible at the base of the epidermis (Figure 5E, insert). In the 7–8 week old embryo, the muscular fibers are arranged in a regular pattern of ring musculature and longitudinal muscle (Figure 5F). The epidermis of the embryo is now clearly organized into the outer cells of the integument, cylindrical epithelial cells, and basiepidermal neurons (Figure 5F’). Between the cylindrical cells, we observe smaller cells with extensions to the nerve net that are likely sensory cells of the epidermis (Figure 5F’). In the juvenile, the sub-epidermal muscular network is now more prominent and forms a muscular sheath surrounding the internal region. The juvenile also has a well-developed basiepidermal neural network, however we could not detect any nerve condensations or indications of the forming digestive system.

### Anatomy of the hatchling

**Figure 6:**
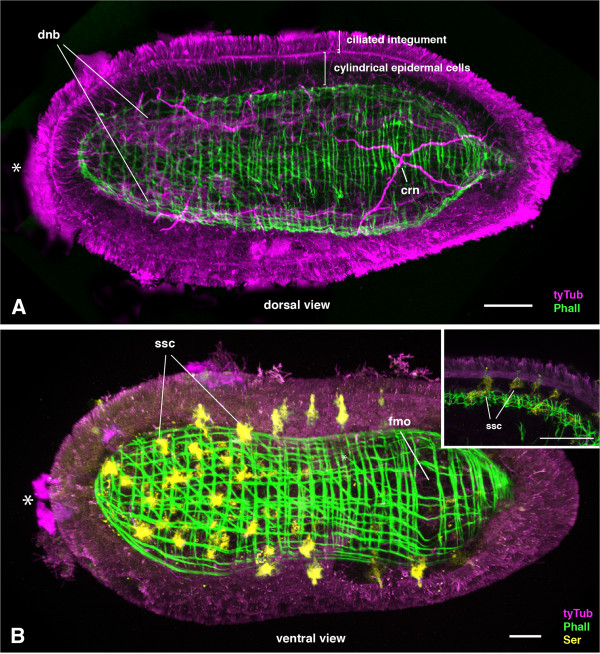
***Meara stichopi *hatchlings**, **general morphology and serotonergic cells.** Optical stacks of different juveniles labeled with antibodies and BODIPY FL-Phallacidin. Anterior is indicated with an asterisk. **A)** Dorsal view of hatchling labeled with anti-tyrosinated tubulin antibody (magenta) and BODIPY-phallacidin (green). The basiepidermal nerve net is located just above the ring and longitudinal musculature of the juvenile. Two bilateral neurite bundles (dnb) are extending from anterior to the posterior along the body with a more anterior concentration of axon tracks. A prominent cross nerve (crn) is visible more posterior. The musculature is forming a spindle-shaped sheath around the body and is composed out of ring musculature and longitudinal muscles. **B)** Ventral view of hatchling of *Meara stichopi* labeled with anti-tyrosinated tubulin antibody (magenta), BODIPY FL-phallacidin (green) and anti-serotonin antibody (yellow). The location of the future mouth is indicated (fmo), but the mouth is not formed yet. The anti-serotonin antibody is labeling cells that are located in the epidermis on the ventral side of the animal. The shape of these cells is indicating a sensory function and a higher concentration of these cells is found anterior. Similar sensory cells are also found on the dorsal side of the hatchling (not shown). The inlet shows a close up of an optical section of the hatchling. The epidermal serotonergic sensory cells (ssc) are directly connected to the muscular system and possess extensions to the outer epidermis. Scale bar 15 μm

**Figure 7:**
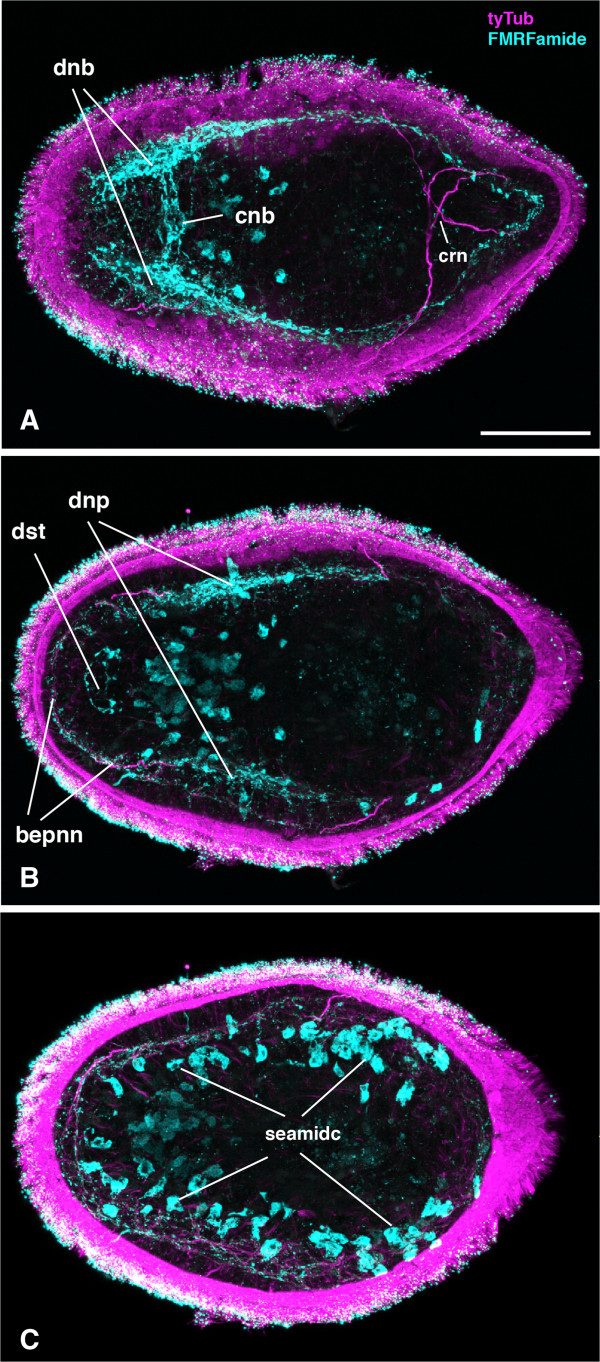
**Morphology hatchlings of *Meara stichopi*: FMRFamide signal.** Different optical sections through a hatchling of *Meara stichopi* labeled with anti-tyrosinated tubulin (magenta) and anti-FMRFamide (cyan) antibodies, anterior to the left. **A)** Dorsal section shows neurite bundles (dnb). A basiepidermal ‘commissural’ neurite bundle (cnb) is connecting the two bilateral longitudinal bundles. The longitudinal neurites extend to the posterior end, where the two strands are connected. The dorsal crossing nerves are visible (crn). **B)** More ventral optical section of the confocal stack. The basiepidermal nerve net (bepnn) is visible and FMRFamide-signal is detected internally around the double statocyst. **C)** Ventral optical section of the same hatchling as in **A)** and **B)**. Subepidermal cells that are labeled with the anti-FMRFamide antibody are visible (seamidc). The nature of these cells remains unclear. Scale bar 20 μm.

After 9–10 weeks of development, the hatchling emerges from the eggshell. The juvenile worm is slightly larger than the length of the eggshell (approximate 100 μm). The characteristic double-statocyst is clearly visible in the hatchling and the major parts of the nervous system are established (Figure 6). We could not detect any epithelia of the digestive system in the juvenile nor is a mouth opening present (Figure 6). The juvenile epidermis is composed of flat, multiciliary integument cells (Figure 6A, Additional file 1A) that cover a thicker layer composed of cylindrical and sensory cells (Figure 6A). At the base of the epidermis, a dense network of axon tracts extends through different regions of the body (Figure 6A). On the dorsal side of the juvenile, multiple axon tracts are bundled into two bilateral condensations that extend from anterior to posterior (Figure 6A). These bundles are broader at the anterior end and are connected by a commissural bundle (Figure 6A). These bundles are anlage of the more prominent basiepidermal dorsal nerve condensations of the adults. At the posterior region, nerves cross in the median of the body (Figure 6A; Figure 7A), a feature observed in both juveniles and adults.

On the ventral side, no such condensations of axon tracts are observed (Figure 6B). Serotonin-positive sensory cells are located in the epidermis, and are connected to the basiepidermal nerve net and possess extensions through the layer of ciliated cells (Figure 6B inlet, Additional file 1H, I). There are more serotonergic cells detected in the anterior ventral region than in the posterior regions and the dorsal side (Figure 6B). The nervous system of the *M. stichopi* juvenile appears to have some specialized neurons, as there is a subset of FMRFamide positive neurons within the basiepidermal anterior bundles and commissure (Figure 7, Additional File 1B-F). Additionally, there are serotonin positive sensory cells, including axon tracts in the anterior region (Additional file 1 H, I). Since the statocyst is located internally, below the muscle sheet, axon tracts connect the cells of the double-statocyst to the basiepidermal nerve net (Additional file 1I). The statocyst is also connected to the muscle sheet (Additional file 1G). It is likely that these muscles help to keep the statocyst in place. In addition to the FMRFamide-positive cells of the dorsal neural bundles, we also detect positive cells that are more ventrally and internally located, whose function remains unknown (Figure 7A-C).

**Figure 8:**
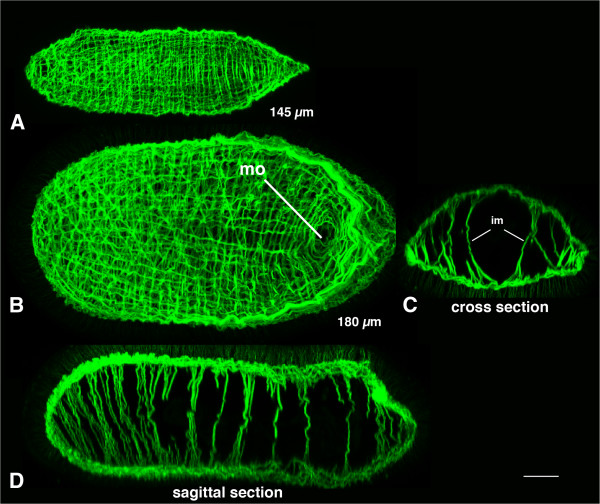
**Musculature of hatchlings of *Meara stichopi*.** Musculature of two different stages of *Meara stichopi* juveniles, anterior to the left. **A)** Ventral view on a juvenile that hatched in the laboratory. The muscle sheath is surrounding the whole body and no mouth opening is formed yet. **B)** A ventral view on a larger and older juvenile collected from the gut of the sea cucumber with the mouth opening (mo) present. **C)** Optical cross-section through the animal shown in **B)**. Internal muscle strands (im) extend from the dorsal to the ventral side. **D)** Longitudinal optical section through juvenile shown in **B)**. The dorso-ventral internal muscle is arranged along the anterior-posterior axis in a serial fashion. Scale bar 10 μm.

The muscle sheet of the hatchling is regular and composed of ring musculature and longitudinal musculature. No mouth opening is visible in the hatchlings (Figure 8A), which is similar to the juveniles of *Nemertoderma westbladi*[23]. All juveniles collected from the gut of the sea cucumber had a mouth opening (Figure 8B), so this could be either be due to progressing differentiation or an inductive effect by the sea cucumber. The optical cross sections indicate that the muscle fibers connect the dorsal and ventral musculature (Figure 8C). This dorso-ventrally arranged musculature follows a regular pattern along the anterior-posterior axis of the juvenile (Figure 8D).

## Discussion

### A reconstruction of the life cycle of *Meara stichopi*

Our samplings and observations show that *M. stichopi* has an annual life cycle that is strongly connected to the host sea cucumber (Figure 2E). Our collections allow us to reconstruct that upon entering the foregut of the sea cucumber, individuals grow inside the host until the reproductive phase in August-October. After depositing the eggs, the adults are then digested by the host. The embryos possess a tough eggshell that probably allows them to exit the gut of the sea cucumber unharmed. Embryogenesis and early postembryonic development takes up to three months and likely happens in the muddy sediment during winter. The hatchlings seem to survive on the remaining yolk until they are taken up by the sea cucumbers in January-March (Figure 2E). We also observed in November and December that the gut of the sea cucumbers is mostly empty of food, and gut parasites, such as the gastropod *Enteroxenos*, which infest the host. Although first described as a ‘parasite’, Westblad [20] considered *M. stichopi* to be commensal because if there is damage to the host, it is only minimal. Our findings that following the reproductive phase, adults even get digested by the host, suggesting that the impact of *M. stichopi* on the sea cucumber is even less than previously assumed. The possible loss of energy is confined to the homeostasis of the individual worms and to the yolk deposition into the eggs that leave the sea cucumber.

### The development and architecture of the nervous system

The nemertodermatid nervous system has previously been investigated using histological [20,21] and immunocytochemical [24,25] methods and is described as entirely basiepidermal. Unlike acoels, nemertodermatids have no portions of the nervous system internalized in a way that they are located below the muscle sheath. The exception is the innervation of the statocyst, which is connected via nerve fibers to the outer basiepidermal plexus. There are no brain-like structures described for nemertodermatids – the anterior condensations are exclusively basiepidermal and ring-shaped (*Nemertoderma westbladi*[24,25]) or just connected by a commissure composed out of neurite bundles (*Meara stichopi*[25]). Our results confirm this structure for *Meara stichopi* and show that the dorsal neurite bundles persist from an anlage in the hatchling to the fully formed structure in the adult. The use of the tyrosinated-tubulin antibody reveals the presence of a larger net of neurons that extend axon tracts also to the internal of the body, while just a subset is stained by the anti-serotonin and anti-FMRFamide antibodies. The dorsal anlage of the two bilateral, longitudinal, thickenings of the nerve plexus are wider than previously described, with a more prominent anterior thickening. Interestingly, such dorsal longitudinal condensations are not found in *Nemertoderma westbladi*, which instead has a pair of ventral and lateral condensations [24]. A previous study by Raikova et al. [25] describes the presence of ‘parenchymal fibre bundles’ in *M. stichopi*. Our results using anti-tyrosinated, anti-FMRFamide and anti-serotonin antibodies, along with BODIPY FL-phallacidin, shows that these ‘fibre bundles’ are basiepidermal, located above the muscle sheet and not internally. Contrary to previous observations [24,25], we have detected positive immunoreactivity around the statocyst using anti-serotonin and anti-FMRFamide antibodies (Additional file 1B-F). Axon tracts connect the statocyst to anterior epithelial cells and to the dorsal basiepidermal nerve condensations. In accordance with previous reports, we could not detect any stomatogastric nervous system in the juvenile of *M. stichopi*. The nervous system of *M. stichopi*, as well as that of other nemertodermatids, is devoid of any prominent internalized structures, such as brains or neurite bundles, which are present in some acoel groups. The nervous system of nemertodermatids is more similar to the nervous system of xenoturbellids, which lacks condensations and only consists of a basiepidermal nerve plexus [26]. Recent phylogenomic analyses [9,10,13] suggest that *Xenoturbella* is closely related to the Acoelomorpha (Xenacoelomorpha). Since Xenoturbella and nemertodermatids both lack subepidermal condensations, this condition has to be considered as plesiomorphic for the whole group and the internalized brain and neurite bundles (‘cords’) found in some acoel taxa have been secondarily evolved from a basiepidermal nerve net. This interpretation hinges on the phylogenetic position of the Xenacoelomorpha as a whole. In the case of Xenacoelomorpha within the Deuterostomia [10], multiple losses of brain-like and cord-like structures in the Xenacoelomorpha must be considered. However, it is difficult to explain why some lineages display only dorsal condensations (*M. stichopi*), and some lineages only ventral and lateral condensations (*Nemertoderma*) [24], as remnants of an ancestral ventrally condensed nervous system. Further molecular studies are necessary to place the Acoelomorpha in the animal tree of life and to clarify the homology of specific substructures found in this fascinating group of animals.

### Comparison of the development with *Nemertoderma westbladi* and acoels

**Figure 9:**
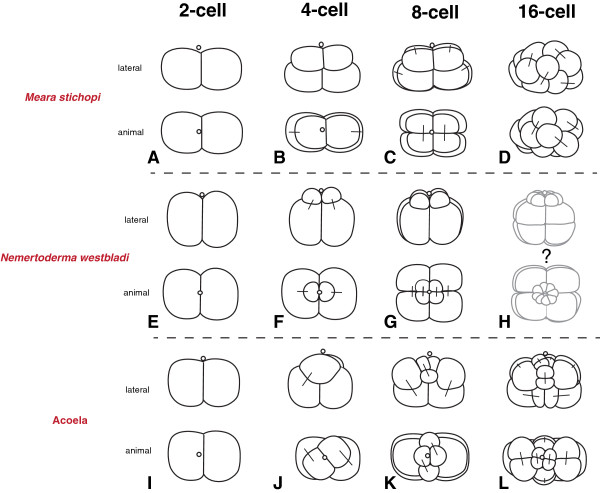
**Schematic drawings of the comparisons of early acoelomorph embryos up to the 16-cell stage.** Comparison between the development up to the 16-cell stage between the nemertodermatids *M. stichopi ***A)** 2-cell **B)** 4-cell, **C)** 8-cell **D)** 16-cell stage and previously described *N. westbladi* (**E-F**, same arrangement of the stages as for *M. stichopi*) and acoel embryos (**I-L**, same arrangement of the stages as for *M. stichopi*) (see discussion in the text). The 16-cell stage of *N. westbladi* is shaded and labeled with a question mark because it has not been documented in [22] with photographs and own observations could not confirm this blastomere arrangement. Bars connect sister blastomeres in all stages.

Studies of acoel development describe a characteristic ‘duet-cleavage’ for all investigated species so far [27-33] (Figure 9I-L). In the ‘duet-cleavage’ program, the blastomeres of the 2-cell stage give off two smaller micromeres to the animal pole (Figure 9J) The embryo thus has one ‘duet’ of micromeres at the animal pole and two macromeres at the vegetal pole. This arrangement of blastomeres in the 4-cell stage is similar between the acoel and the nemertodermatid embryos studied so far and can be interpreted as an apomorphy for the Acoelomorpha (Figure 9B, F, J). The following round of divisions differs between the nemertodermatid and the acoel embryo: in the acoel embryo, the vegetal macromeres divide again equatorially and unequally (Figure 9K), while in both nemertodermatid species the macromeres divide meridional and equally (Figure 9C, G). In the acoel embryo, the cleavage plane is shifted in an angle of about 45 degrees to the animal-vegetal axis of the embryo (Figure 9K), while in both nemertodermatid species the cleavage plane of the micromeres is strictly meridional (Figure C, G). The acoel cleavage program differs significantly from our present description of *M. stichopi* and the previous description of the cleavage of *N. westbladi*[22] (Figure 9). The only unequal division observed in both nemertodermatid species is the 2^nd^ cell division (Figure 9B, F), while the last unequal division in the acoel is described in the 3^rd^ division of the two vegetal macromeres (Figure 9L). Acoel embryos possess a more stereotypic arrangement of blastomeres up to the 32-cell stage (see for example Gardiner [30]). In acoels, two vegetal macromeres will gastrulate and form the entire endomesoderm of the embryo. These two cells gastrulate during the transition of the 12-cell to the 24-cell stage [29,30,34]. In *M. stichopi*, gastrulation happens one to two cell cycles later, between the 24-cell and 64-cell stage. The nemertodermatid pattern of 4 vegetal macromeres and 4 animal micromeres is reminiscent of the 8-cell stage of a spiralian embryo, although it is formed in a completely different way.

Although the general pattern of the first divisions of the *M. stichopi* embryo is similar to the cleavage of *N. westbladi*[22], the major differences are the more spherical shape of the *N. westbladi* embryo versus the oval shape of the *M. stichopi* embryo and the considerable size differences between the micromeres (Figure 9A-H). The later development of *M. stichopi* is characterized by an inner cell mass of large, equal-sized blastomeres, which are surrounded by a monolayer of smaller blastomeres. A similar pattern is also present in acoel embryos [6,29,30,33]. The first structure that emerges in acoelomorph embryos is the muscular grid that can be identified by fluorescently labeled phallotoxins [33] (Figure 5). In the acoel *Isodiametra pulchra*, the musculature starts to form at the animal pole (=anterior) of the embryo and progresses to the posterior end of the embryo [33]. In contrast, no such gradient is present in *M. stichopi*, as the musculature appears simultaneously along the entire body axis. Similar to *I. pulchra*, the ring musculature of *M. stichopi* is formed before the longitudinal musculature and both are formed before elements of the nervous system are detectable. The formation of the muscular sheath coincides with the differentiation of the outer epidermis and the formation of the cilia (Figure 5). Since the nervous system of *M. stichopi* is basiepidermal, one should not expect epidermal cells to immigrate internally below the muscle sheet. An exception might be the statocyst sensory complex at the anterior end, but its formation remains unclear. This is different from the nervous system development in acoels where the nervous system is formed by all micromeres [32] and cells from the outer sheet migrate to form neural structures [35].

## Conclusions

The nemertodermatid *Meara stichopi* has an annual life cycle with a main reproductive period inside the foregut of the holothurian *Parastichopus tremulus*. The development of the embryos undergoes an early stereotypic cleavage, and embryogenesis takes 9–10 weeks until the juvenile hatches. The cleavage program of *M. stichopi* shows significant differences to that of the sister group, Acoela. The musculature is formed before the nervous system, similar to what has been described in acoel embryos. Our study demonstrates the variability of the development in the Acoelomorpha and that further studies are needed to reconstruct the ground pattern of acoelomorph development.

## Methods

### Collection and maintenance of *Meara stichopi*

Sea cucumbers of the species *Parastichopus tremulus* (Gunnerus, 1767), the host of *Meara stichopi*, were collected throughout the year between Winter 2009/2010 – Winter 2013/2014 at collection sites around Bergen, Norway. Between 10 – 50 sea cucumbers were collected from 200 – 350 m depth using the “Schander Sled” (Figure 1) during each collection trip and brought to the lab at the Sars Centre for dissection. Sea cucumbers were opened and the digestive tracts were examined for the presence of *M. stichopi*. Approximately 3000 *M. stichopi* have been collected in total and about 800 individuals deposited a total of about 2500 embryos during the reproductive seasons. Juveniles and adults of *M. stichopi* were transferred to filtered seawater and kept at 5–8 degrees in glass bowls. Seawater was changed every 2–3 days and animals were kept for up to 2–3 months in the lab. Gravid adults deposited oocytes into bottoms of the glass bowls in a jelly-mesh and sperm was surrounding the eggs (Figure 1). Fertilized eggs were cultured in petri dishes containing seawater supplemented with Penicillin (100 Units/ml) and Streptomycin (100 μg/ml) and kept at 5–8 degrees.

Collection spots:

Hauglandsosen (60 24.533 N 5 06.566 E).

Lysefjorden (60 12.347 N 05 17.903 E).

Hjeltefjorden (60 24.366 N 05 06.111 E).

Raunefjorden (60 15.896 N 05 08.448 E).

Laboratory work on the species *M. stichopi* does not raise ethical issues. Therefore approval from a research ethics committee is not required.

### Egg shell penetration and fixation of embryos and adults

Egg shells of embryos and pre-hatchlings were penetrated using 1% Thioglycolate/0.05% Pronase (Sigma Aldrichs 5147) in sea water (pH 8.0) for 4 hours at 4°C. During development, the eggshell extends slightly along the long axis and softens, such that late stage embryos are easier to penetrate with a needle than early cleavage stages. Before fixation, embryos, juveniles and adults were relaxed with 7.5% MgCl_2_ in Millipore water, and fixed using 4% Paraformaldehyde in filtered sea water for 1 hour at 4°C. Fixed specimens were washed four times in PBS containing 0.1% Triton X (PTx) and stored at 4°C before subsequent staining.

### Antibody and phallacidin staining

Before the antibody staining, holes were poked into the eggshell of the fixed embryos with insect pins to facilitate the penetration of the antibodies (total n = 500). Antibodies against tyrosinated tubulin (Sigma) and BODIPY®FL labeled phallacidin (Molecular Probes) were used to label the embryos and juveniles following a standard procedure [36]. The phallacidin was used to visualize the F-actin of the cell cortex and the muscle fibers of the embryo, however, it also labeled the centrosomes of some early embryos (e.g. 7 day old, Figure 4D). Anti-serotonin (Sigma) and anti-FMRFamide (Sigma) antibodies were used to label substructures of the nervous system. Specimens were blocked with two 15 min washes in PTx + 0.1% BSA (Bovine Serum Albumin) followed by a 30 min incubation in PTx + 5% normal goat serum. Specimens were incubated with the primary antibody (mouse anti-tyr-tub 1:500, rabbit anti-serotonin 1:200, rabbit anti-FMRFamide 1:200) in PTx + 5% goat serum overnight at 4°C on a shaker. Primary antibody was removed with three 5 min and four 30 min washes in PTx + BSA and an additional blocking step in PTx + normal goat serum for 30 min. Specimens were incubated with the secondary antibody (Cy3 labeled anti-mouse IgM and Cy5 labeled anti-rabbit IgM) diluted 1:200 in PTx + normal goat serum overnight. The secondary antibody was removed with three 5 min and four 30 min washes in PTx + BSA. BODIPY FL phallacidin was added to some samples by first washing the specimens in PBS and incubating in 3–10 Units/ml PTx for 2 hours. Specimens were then washed three times in PBS and prepared for mounting. Propidium Iodide was used to stain the nuclei in some of the samples following a standard protocol in which 0.01 mg/ml propidium iodide was added to the incubation with BODIPY FL-phallacidin. 

### Confocal microscopy

Specimens were mounted in ‘Murray’s Clear’ (2:1 mixture of benzyl benzoate and benzyl alcohol). Prior to transfer to Murray’s Clear, specimens were subjected to a series of isopropanol washes (70%, 85%, 95%, 100%). Specimens were imaged using a Leica SP5 confocal microscope. Image stacks were rendered using Imaris 7.6 (Bitplane).

### 4D-microscopy

Embryos were recorded using a 4D-microscopy system (modified system after Hejnol & Schnabel [37]). Zygote and 2-cell stages were mounted in seawater, covered with a coverslip and sealed with Vaseline. Recordings (n = 3) were conducted at 10°C and Z-stacks composed out of 50 images were taken every 10 minutes. Cells were traced using the software SIMI°BioCell.

### Documentation

Images of juveniles and adults were taken using a Canon 5D Mark III mounted on a Leica 120 M dissecting scope or with a Zeiss AxioCam HRc mounted on a Zeiss Axio Skope.A1.

## Competing interest

The authors declare that they have no competing interests.

## Authors’ contributions

AB carried out the confocal studies and edited the manuscript. AH designed the study and conducted the 4D-microscopic analysis, 3D-reconstruction of the confocal data and analysis of the data and wrote the manuscript. AB and AH collected the animals, cultured the embryos and conducted the labeling and documentation. Both authors read and approved the final manuscript.

## Supplementary Material

Additional file 1 **Details of *M. stichopi *** **hatchling.** A) Epidermis cells of the integument of a *M. stichopi* juvenile labeled with anti-tyrosinated-tubulin. B) Optical section showing the FMRFamidergic innervation (arrowheads) of the statocyst. C) anti-FMRFamide (cyan) and anti-tyrosinated tubulin labeling (magenta) of a anterior part of a hatchling. The signal of the anti-FMRFamide antibody shows neurons of the anterior, dorsal basiepidermal nerve condensations. The bilateral neurite bundles are connected with a commissural neurite bundle (cnb) D) Sagittal optical section through a hatchling labeled with anti-tyrosinated tubulin (magenta) and anti-FMRFamide (cyan). Subepidermal cells (seamidc) of unknown function are labeled with the anti-FMRFamide antibody. E) Optical cross section through same juvenile as B, C and D showing FMRFamidergic neurons (arrowheads) connecting the statocyst with the basiepidermal dorsal nerve condensations (dnc). F) Optical cross section through juvenile showing ventral FMRFamide positive cells (magenta, arrows) that are below the epidermis (red label, anti-tyrosinated tubulin). G) Optical section of the anterior part of a hatchling showing the position of the subepidermal statocyst (dst). Four statocyst muscles (stm) are connected to the cells around the statocyst. H) Optical section showing the innervation of statocyst (dst) by serotonergic neurons (yellow). I) Hatchling labeled with anti-tyrosinated tubulin (magenta), BODIPY-FL phallacidin (green) and anti-serotonin (yellow). Optical section showing the innervation of statocyst (dst) by serotonergic neurons (yellow). The statocyst is internal from the muscle sheath (BODIPY FL-phallacidin, green), Scale bar 15 μm, anterior is indicated with an asterisk. Click here for file
