# Supplementary figures and images for: Development and juvenile anatomy of the nemertodermatid Meara stichopi (Bock) Westblad 1949 (Acoelomorpha)
Source: Front Zool. 2014 Jul 7;11:50. doi: 10.1186/1742-9994-11-50 (PMC4094782; doi:10.1186/1742-9994-11-50)

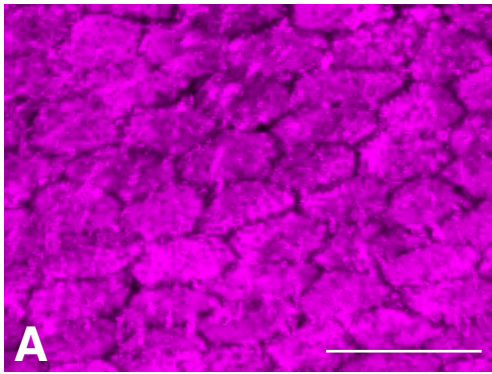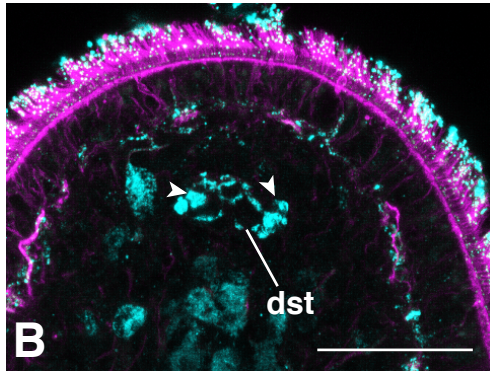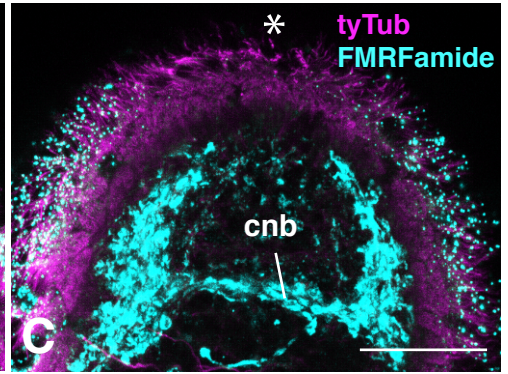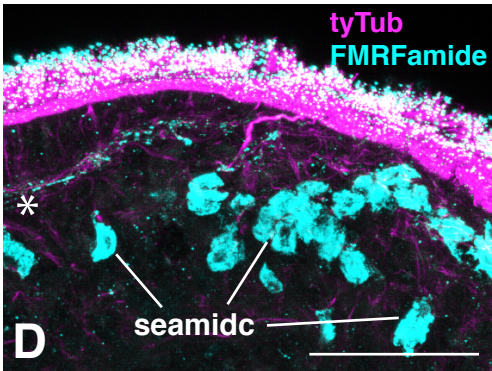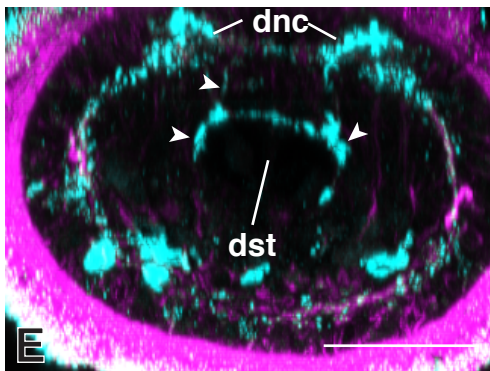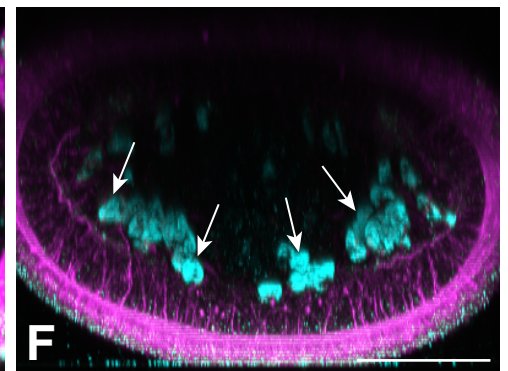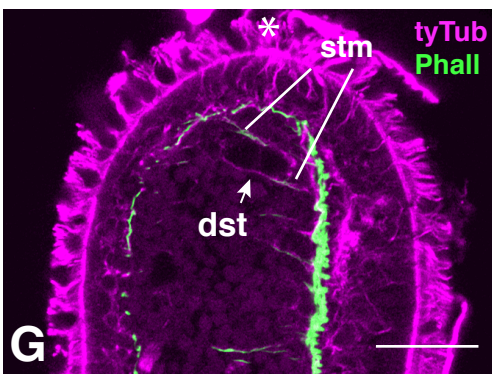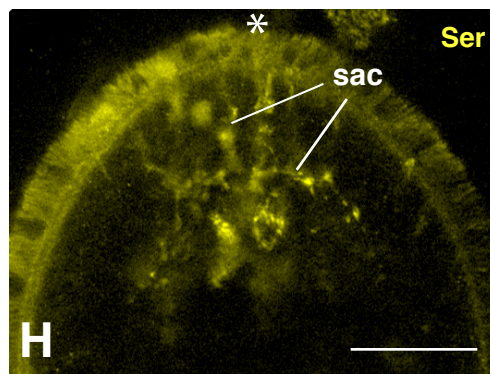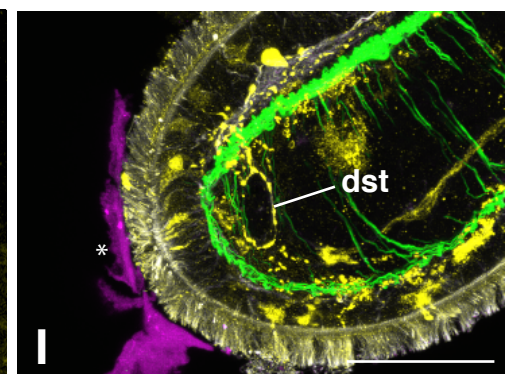

Supplement: Additional file 1 — Details of M. stichopi hatchling. A) Epidermis cells of the integument of a M. stichopi juvenile labeled with anti-tyrosinated-tubulin. B) Optical section showing the FMRFamidergic innervation (arrowheads) of the statocyst. C) anti-FMRFamide (cyan) and anti-tyrosinated tubulin labeling (magenta) of a anterior part of a hatchling. The signal of the anti-FMRFamide antibody shows neurons of the anterior, dorsal basiepidermal nerve condensations. The bilateral neurite bundles are connected with a commissural neurite bundle (cnb) D) Sagittal optical section through a hatchling labeled with anti-tyrosinated tubulin (magenta) and anti-FMRFamide (cyan). Subepidermal cells (seamidc) of unknown function are labeled with the anti-FMRFamide antibody. E) Optical cross section through same juvenile as B, C and D showing FMRFamidergic neurons (arrowheads) connecting the statocyst with the basiepidermal dorsal nerve condensations (dnc). F) Optical cross section through juvenile showing ventral FMRFamide positive cells (magenta, arrows) that are below the epidermis (red label, anti-tyrosinated tubulin). G) Optical section of the anterior part of a hatchling showing the position of the subepidermal statocyst (dst). Four statocyst muscles (stm) are connected to the cells around the statocyst. H) Optical section showing the innervation of statocyst (dst) by serotonergic neurons (yellow). I) Hatchling labeled with anti-tyrosinated tubulin (magenta), BODIPY-FL phallacidin (green) and anti-serotonin (yellow). Optical section showing the innervation of statocyst (dst) by serotonergic neurons (yellow). The statocyst is internal from the muscle sheath (BODIPY FL-phallacidin, green), Scale bar 15 μm, anterior is indicated with an asterisk. [file 1742-9994-11-50-S1.pdf]
